# Macrophage Polarisation: an Immunohistochemical Approach for Identifying M1 and M2 Macrophages

**DOI:** 10.1371/journal.pone.0080908

**Published:** 2013-11-15

**Authors:** Mário Henrique M. Barros, Franziska Hauck, Johannes H. Dreyer, Bettina Kempkes, Gerald Niedobitek

**Affiliations:** 1 Institute for Pathology, Unfallkrankenhaus, Berlin, Berlin, Germany; 2 Institute for Pathology, Sana Klinikum Lichtenberg, Berlin, Germany; 3 Department of Gene Vectors, Helmholtz Zentrum, National Research Center for Environmental Health, Munich, Germany; University Hospital Freiburg, Germany

## Abstract

Macrophage polarization is increasingly recognised as an important pathogenetic factor in inflammatory and neoplastic diseases. Proinflammatory M1 macrophages promote T helper (Th) 1 responses and show tumoricidal activity. M2 macrophages contribute to tissue repair and promote Th2 responses. CD68 and CD163 are used to identify macrophages in tissue sections. However, characterisation of polarised macrophages *in situ* has remained difficult. Macrophage polarisation is regulated by transcription factors, pSTAT1 and RBP-J for M1, and CMAF for M2. We reasoned that double-labelling immunohistochemistry for the detection of macrophage markers together with transcription factors may be suitable to characterise macrophage polarisation *in situ*. To test this hypothesis, we have studied conditions associated with Th1- and Th2-predominant immune responses: infectious mononucleosis and Crohn’s disease for Th1 and allergic nasal polyps, oxyuriasis, wound healing and foreign body granulomas for predominant Th2 response. In all situations, CD163+ cells usually outnumbered CD68+ cells. Moreover, CD163+ cells, usually considered as M2 macrophages, co-expressing pSTAT1 and RBP-J were found in all conditions examined. The numbers of putative M1 macrophages were higher in Th1- than in Th2-associated diseases, while more M2 macrophages were seen in Th2- than in Th1 related disorders. In most Th1-related diseases, the balance of M1 over M2 cells was shifted towards M1 cells, while the reverse was observed for Th2-related conditions. Hierarchical cluster analysis revealed two distinct clusters: cluster I included Th1 diseases together with cases with high numbers of CD163+pSTAT1+, CD68+pSTAT1+, CD163+RBP-J+ and CD68+RBP-J+ macrophages; cluster II comprised Th2 conditions together with cases displaying high numbers of CD163+CMAF+ and CD68+CMAF+ macrophages. These results suggest that the detection of pSTAT1, RBP-J, and CMAF in the context of CD68 or CD163 expression is a suitable tool for the characterisation of macrophage polarisation *in situ*. Furthermore, CD163 cannot be considered a reliable M2 marker when used on its own.

## Introduction

Macrophages are increasingly recognised to represent heterogeneous cell populations with functional variability depending on polarization status [1–5]. As yet, two differentiation patterns, termed M1 and M2, have been characterised. M1 macrophages are characterized by a pro-inflammatory phenotype, promotion of T helper 1 (Th1) immune response and tumoricidal activity while M2 macrophages display regulatory functions in tissue repair, remodelling and promotion of Th2 immune response [2–4]. M1 and M2 macrophages are distinguished by the differential expression of diverse molecules, e.g., iNOS, metalloproteinases and arginase [1]. However, none of these antigens is suitable for single-marker identification of polarised macrophages by immunohistochemistry. Based on *in vitro* studies, it has been suggested that CD163 may be a M2 marker [5–8] and studies of human tissues, e.g., in cancer research, have considered CD163+ cells identified by immunohistochemistry as M2 macrophages [9–14]. However, it is an open issue if all CD163+ cells present in the tissue microenvironment represent M2 macrophages [15]. 

Additionally, studies focussing on macrophage polarization are mostly performed *in vitro* and do not reflect the complexity of immune responses observed *in vivo* [5,16]. Thus, *in situ* characterisation of macrophage polarisation is an important issue.

Several studies have identified transcription factors directing macrophage polarisation. STAT1 is upregulated in response to types I, II or III interferons and its phosphorylated form (pSTAT1) binds to the promoter region of interferon-stimulated genes [4,17]. CMAF is an essential transcription factor for interleukin (IL) -10 gene expression in macrophages [18]. We, therefore, hypothesised that the combined detection of a macrophage-specific marker, such as CD68 or CD163, together with pSTAT1 or CMAF might be used to identify M1 or M2-polarized macrophages [19,20]. Recently, it has been described that Notch signalling determines M1 polarization of macrophages and that RBP-J is an important mediator of this signalling pathway [21,22]. This raises the possibility that detection of nuclear RBP-J in macrophages may be another marker for M1 polarisation.

The objectives of this study were to evaluate if a double-staining immunohistochemistry approach combining generic macrophage markers such as CD68 and CD163, with antibodies specific for pSTAT1, CMAF or RBP-J can be used to evaluate macrophage polarization in human tissues, and if CD163 is a specific M2 marker *in vivo*.

## Materials and Methods

### Tissues

Formalin-fixed paraffin-embedded (FFPE) tissue blocks from 68 cases were included in this study. These included 17 tonsils with a diagnosis of acute infectious mononucleosis (IM) and eleven cases of Crohn´s disease (CD) representing diseases with a predominant cytotoxic/Th1 immune response [23,24]. As Th2 immune response models, 11 cecal appendices with oxyuriasis [25], 10 allergic nasal polyps with prominent eosinophilia [2,16], 10 skin biopsy samples showing wound healing [26,27] and 9 skin samples with foreign body granulomas were included [28]. All cases were selected from the archives of the Institute of Pathology, Unfallkrankenhaus Berlin. All materials were submitted for diagnostic or therapeutic purposes and were used in accordance with national ethical principles. No tissue samples have been collected solely for the purpose of this study. All histological diagnoses were reviewed before inclusion in this study.

### Double staining immunohistochemistry

Whole tissue sections were used in all cases. For immunohistochemistry, blocks were sectioned at a thickness of 3 μm. Buffers used for antigen retrieval and primary antibodies are listed in the Supporting Information (Table S1). Briefly, the following antibody reagents were used: CD68 (clone PG-M1, Dako), CD163 (clone 10D6, Novocastra), anti-pSTAT1 (polyclonal, Santa Cruz), 1anti-CMAF (clone M-153, Santa Cruz) and anti-RBP-J (clone RBP 3E2, which was produced as described [29]). 

Antigen retrieval was performed by heat treatment in a pressure-cooker for 1 minute with HIER T-EDTA Buffer (Zytomed Systems, Berlin, Germany). After incubation with appropriately diluted pSTAT1-, RBP-J- or CMAF-specific reagents (30 minutes), immobilized antibodies were detected using ZytoChem Plus HRP polymer kit (Zytomed Systems, Berlin, Germany) (Supporting Information, Table S1), employing diaminobenzidine (DAB) chromogen as substrate. Subsequently, slides were washed in Wash Buffer (Zytomed Systems, Berlin, Germany) for 5 minutes and the appropriately diluted CD68 or CD163 antibodies was incubated overnight at 4°C. Following another washing step using Wash Buffer (Zytomed Systems, Berlin, Germany), bound antibodies were detected using the AP Polymer System (Zytomed Systems, Berlin, Germany), employing Blue Alkaline Phosphatase (Vector Laboratories, California, USA) as substrate. The sections were not counterstained.

For each case, the same area was evaluated for all marker combinations (CD68/pSTAT1, CD68/RBP-J, CD68/CMAF, CD163/pSTAT1, CD163/RBP-J and CD163/CMAF). 

### Computer Assisted Microscopical Analysis

For the quantitative evaluation, each selected-area was photographed using AxioCam MRc camera (Zeiss, Germany) at a 200x magnification. The numbers of labelled macrophages were determined per 1mm^2^ using the image analysis software HISTO (Biomas, Erlangen, Germany). The 50^th^ percentile was used to categorize the intensity of the infiltration (low vs. high).

### M1 vs. M2 Balance

The absolute numbers per mm^2^ for each marker combination were used to calculate the M1:M2 ratio per case, using the combinations: CD68+pSTAT1+ vs. CD68+CMAF+; CD68+RBP-J+ vs. CD68+CMAF+; CD163+pSTAT1+ vs. CD163+CMAF+ and CD163+RBP-J+ vs. CD163+CMAF+. A polarised response (M1 > M2 or M2 > M1) was defined as one cell population 1.5x higher than the other.

### Statistical Analysis

Fisher’s exact test was used to test association between dichotomous variables, while Mann-Whitney test was used to test association between dichotomous and continuous variables. Spearman´s correlation was used to test association between continuous variables. The measure of discrepancy between observed values was evaluated by R^2^ in a scatterplot graphic.

To estimate the total numbers of CD68 and CD163 macrophages, each macrophage marker (MM) was used separately to calculate the arithmetic mean as follows: [(MM+pSTAT1+ + MM+pSTAT1-) + (MM+CMAF+ MM+CMAF-) + (MM+RBP-J+ + MM+RBP-J-)]/3.

To evaluate the reproducibility of the staining evaluation, 26 cases were randomly selected and all the markers were re-counted by a second investigator (F.H.), using the same criteria as applied by the first pathologist (M.H.M.B.). The sample size was defined according to Walter et al [30]. Two-way random-effect ANOVA was used to calculate the intraclass correlation coefficient (ICC). An ICC between 0.61 and 0.80 was considered as substantial agreement, while an ICC between 0.81 and 1.00 was considered as excellent agreement [31,32]. Specifically for RBP-J, all cases were re-counted by F.H. because of an initial low reproducibility, when only the initial 26 cases were considered. Using this approach, substantial agreement was obtained for the evaluation of CD163+pSTAT1+ (ICC= 0.78; P< 0.0005) and CD163+CMAF+ cells (ICC= 0.69; P< 0.0005); while excellent agreement was achieved for CD68+pSTAT1+ (ICC= 0.82; P< 0.0005) and CD68+CMAF+ cells (ICC= 0.85; P< 0.0005) (Table S1). Following evaluation of all cases, substantial agreement was also achieved for the evaluation of CD163+RBP+ (ICC= 0.75; P< 0.0005) and CD68+RBP+ cells (ICC= 0.73; P< 0.0005) (Table S1).

Hierarchical cluster analysis using average linkage and binary simple matching measure allowed to explore the structure of association among variables of macrophages and Th model diseases. Differences were considered significant at p< 0.05 in 2-tailed tests. Data were analyzed using Statistical Package for the Social Sciences 13.0 (SPSS).

## Results

**Figure 1:**
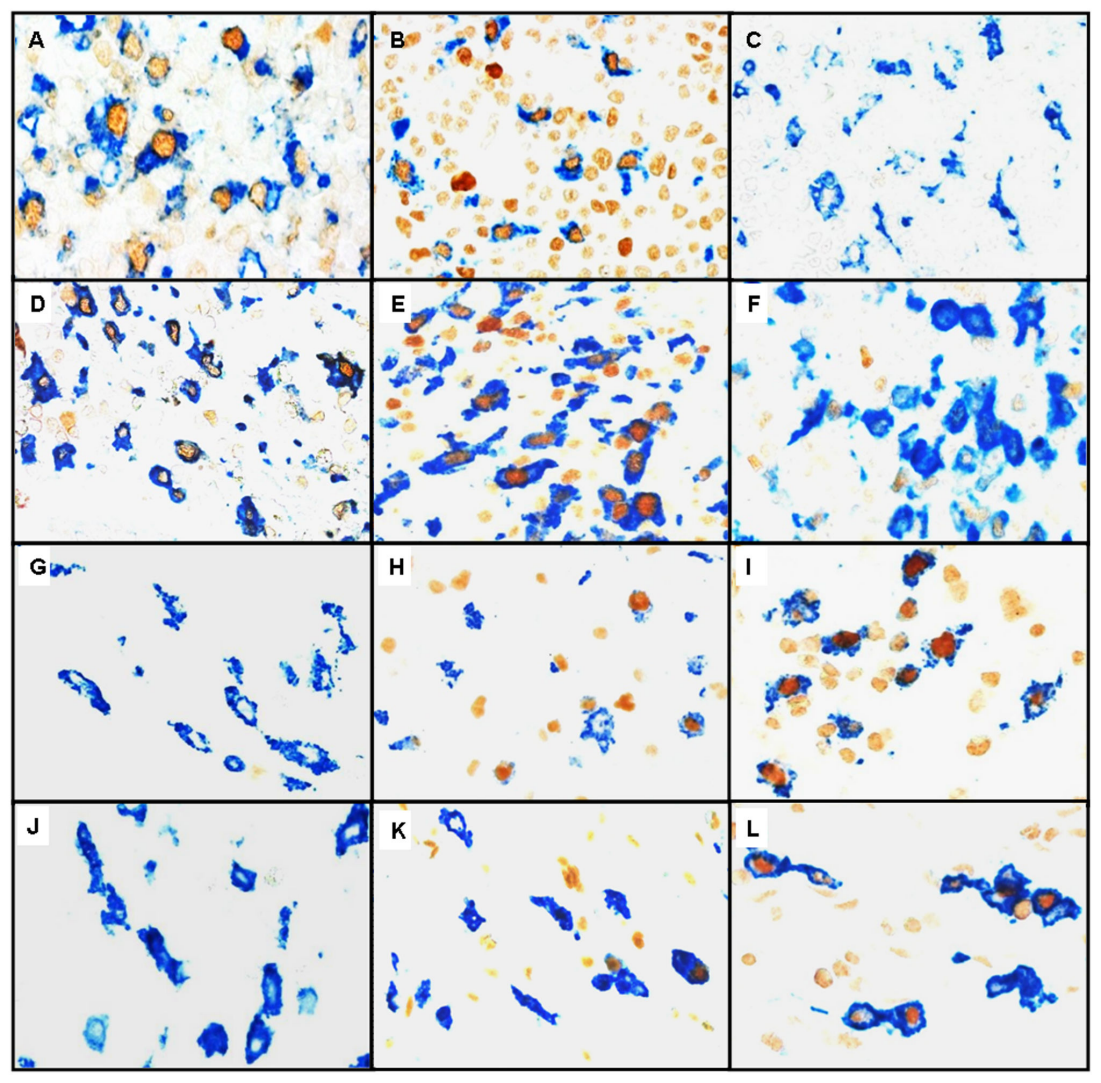
Examples of immunostains used to identify M1 and M2 macrophages. Expression of CD68 or CD168 is indicated by blue cytoplasmic/membraneous staining. The expression of transcription factors pSTA1, RBP-J and CMAF is indicated by brown nuclear staining. (A) Infectious mononucleosis, a Th1 disease, reveals high numbers of CD68+pSTAT1+ macrophages as well as (B) of CD68+RBP-J+ macrophages. By contrast (C) infectious mononucleosis shows, in this field, absence of CD68+CMAF+ macrophages. Similarly, in Crohn’s disease, another Th1-associated condition (D) large numbers of CD163+pSTAT1+ macrophages as well as (E) of CD163+RBP-J+ macrophages are seen. In contrast (F) Crohn´s disease reveals, in this image, absence of CD163+CMAF+ macrophages. (G) An allergic nasal polyp, a Th2-associated disorder, shows, in this field absence of CD163+pSTAT1+ macrophages and (H) rare CD163+RBP-J+ macrophages, while (I) high numbers of CD163+CMAF+ macrophages are present. Similarly, in an allergic nasal polyp (J) in this field there is absence of CD68+pSTAT1+ macrophages and (K) only few CD68+RBP-J+ macrophages are seen. By contrast, (L) high numbers of CD68+CMAF+ macrophages are observed. (original magnification: 400x).

Immunohistochemistry combining CD68 or CD163 with antibodies specific for pSTAT1, RBP-J or CMAF displayed double-positive and single-positive cells in all cases analysed irrespective of histological diagnosis. The distinction between double-positive and single-positive cells was performed easily with little or no background staining (Figure 1). The numbers of each cell population are summarised in the Table 1.

**Table 1:** Number macrophages per mm^2^ according to Th model-diseases.

**DISEASE**	**CD163+ cells /mm^2^ (median)**	**CD163+pSTAT1+ cells /mm^2^ (median)**	**CD163+RBP-J+ cells/ mm^2^ (median)**	**CD163+CMAF+ cells/ mm^2^ (median)**	**CD68+ cells /mm^2^ (median)**	**CD68+pSTAT1+ cells /mm^2^ (median)**	**CD68+RBP-J+ cells/ mm^2^ (median)**	**CD68+CMAF+ cells/ mm^2^ (median)**
**Th1 Model Diseases**	**73 to 369 (178)**	**0 to 272 (80**)	**29 to 261 (79)**	**3 to 202 (46)**	**488 to 251 (151**)	**3 to 190 (97)**	**41 to 179 (108)**	**0 to 96 (18**)
Infectious mononucleosis	116 to 369 (209)	29 to 272 (87)	29 to 131 (79)	14 to 137 (44)	95 to 213 (167)	20 to 190 (108)	41 to 179 (114)	0 to 79 (17)
Crohn´s disease	73 to 255 (138)	0 to 176 (67)	55 to 261 (85)	3 to 202 (49)	88 to 251 (109)	3 to 137 (88)	41 to 149 (79)	8 to 96 (20)
**Th2 Model Diseases**	**49 to 241 (125)**	**0 to 52 (3**)	**3 to 111 (42)**	**20 to 164 (70)**	**34 to 266 (94)**	**0 to 47 (1**)	**5 to 143 (38)**	**11 to 179 (50)**
Foreign body granuloma	96 to 239 (128)	0 to 23 (3)	26 to 111 (38)	23 to 140 (70)	48 to 266 (91)	0 to 14 (1)	5 to 143 (41)	14 to 105 (47)
Wound healing	87 to 210 (160)	0 to 17 (3)	26 to 111 (65)	38 to 164 (92)	61 to 189 (149)	0 to 3 (1)	11 to 99 (42)	35 to 179 (64)
Allergic nasal polyp	49 to 182 (81)	0 to 52 (1)	3 to 82 (24)	20 to 108 (45)	34 to 111 (58)	0 to 29 (1)	5 to 44 (21)	11 to 85 (41)
Oxyuriasis	79 to 241 (127)	0 to 29 (1)	20 to 85 (41)	35 to 135 (70)	73 to 225 (98)	0 to 47 (1)	14 to 102 (49)	38 to 120 (73)

Overall, we observed slightly higher numbers of CD163+ cells than of CD68+ cells (median 140 cells/mm^2^ to CD163 vs. mean 122 cells/mm^2^ to CD68) (Table 1).

Considering infectious mononucleosis and Crohn’s disease as model diseases associated with a predominant Th1 response and oxyuriasis, allergic nasal polyps, wound healing, and foreign body granulomas as Th2-predominant immune responses, we observed higher numbers of CD68+ macrophages in Th1-predominant diseases (median 151 cells/mm^2^) than in Th2-predominant conditions (median 94 cells/mm^2^) (P= 0.001). The same was observed for CD163+ cells (median 178 cells/mm^2^ in Th1 vs. median 125 cells/mm^2^ in Th2; P= 0.002) (Table 1). Within any one Th-predominance group, numbers of CD68+ cells as well as of CD163+ cells varied between individual conditions (Table 1), possibly reflecting differences in tissue composition (e.g. variation in the number of epithelial cells), or differences in the contribution of macrophages in disease pathogenesis.

Next, we analysed the prevalence of putative M1 macrophages (CD68+/pSTAT1+ or CD163+/pSTAT1+) as well as of putative M2 macrophages (CD68+/CMAF+ or CD163+/CMAF+) in different conditions. Furthermore, the prevalence of CD68/RBP-J+ and CD163/RBP-J+ cells was evaluated.

**Figure 2:**
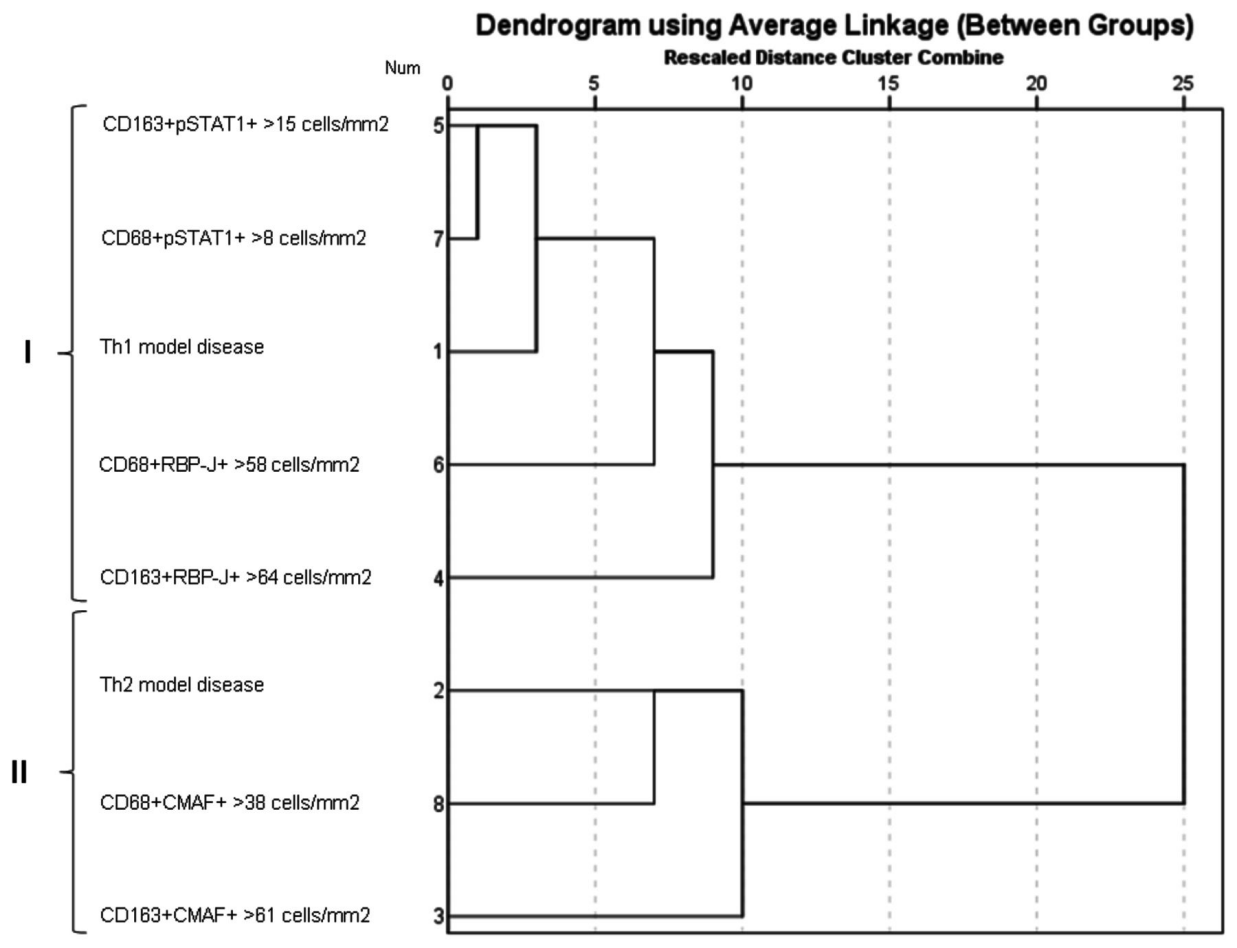
Dendrogram using average linkage obtained by hierarchical cluster analysis. Two main identified clusters (I and II) are identified by brackets. Each cell population is stratified by 50^th^ percentile, indicating high number of this cell in the tissue microenvironment of Th1 and Th2 model diseases. Num: order of variable input.

A preliminary data classification strategy using hierarchical cluster analysis was performed to identify underlying patterns of macrophage polarization (Figure 2). In this analysis, two distinct clusters emerged: cluster I included the diseases characterised by a Th1 immune response together with cases with high numbers of CD163+pSTAT1+, CD68+pSTAT1+, CD163+RBP-J+ and CD68+RBP-J+ macrophages; cluster II comprised conditions with a Th2 immune response together with cases displaying high numbers of CD163+CMAF+ and CD68+CMAF+ macrophages.

Using CD68/pSTAT1 double-staining, variable numbers of CD68+pSTAT1+ and CD68+pSTAT1- macrophages were observed (Table 1). The same was observed in relation to CD163/pSTAT1 double-staining (Table 1). Overall, higher numbers of CD68+pSTAT1+ macrophages were observed in Th1 model diseases (3 to 190, median 97 cells/mm^2^), than in the Th2 model conditions (0 to 47, median 1 cell/mm^2^) (P< 0.0005). Similarly, numbers of CD163+pSTAT1+ macrophages were higher in Th1 model diseases (0 to 272, median 80 cells/mm^2^) than in Th2 model disease (0 to 52, median 3 cells/mm^2^) (P< 0.0005) (Table 1 and Figure 2). 

Furthermore, Th2 model diseases contained higher numbers of CD68+CMAF+ macrophages (from 11 to 179; median 50 cells/mm^2^) than seen in Th1 model disease (0 to 96; median 18 cells/mm^2^) (P< 0.0005). Similarly, higher numbers of CD163+CMAF+ macrophages were observed in the Th2 model diseases (20 to 164; median 70 cells/mm^2^) than in Th1 predominant conditions (3 to 202; median 46 cells/mm^2^), (P= 0.02) (Table 1 and Figure 2). 

Considering the balance between the cell populations, 25 of 28 (89%) Th1 model cases displayed more CD68+pSTAT1+ cells than CD68+CMAF+ macrophages (M1>M2). Two cases of infectious mononucleosis exhibited similar numbers of CD68+pSTAT1+ and of CD68+CMAF+ macrophages (M1 ^≈^ M2) while one case of Crohn’s disease displayed an M2>M1 pattern (Supporting Information, Table S2). In the group of Th1 model conditions, 18 of 28 cases (64%) revealed higher numbers of CD163+pSTAT1+ cells than of CD163+CMAF+ macrophages (M1>M2). Similar numbers of CD163+pSTAT1+ and CD163+CMAF+ macrophages (M1 ^≈^ M2) were observed in seven Th1 cases (3 infectious mononucleosis and 4 Crohn’s disease) while two cases of infectious mononucleosis and one case of Crohn’s disease showed an predominance of CD163+/CMAF+ cells over CD163/pSTAT1+ cells (M2>M1) (Supporting Information, Table S2 and Figure S1). 

In the group of Th2 model conditions, all 40 cases showed an M2>M1 pattern for both CD68 (P< 0.0005) and CD163 (P< 0.0005) (Table 2). 

**Table 2:** Balance of polarized macrophages according to Th model diseases.

**MACROPHAGE BALANCE**	**MODEL DISEASE**		**P**
	**Th1 (%)**	**Th2 (%)**	
**CD163+pSTAT1+ : CD163+CMAF+ cells^a^**			
M1 > M2	18 (64.3)	0	
M2 > M1	3 (10.7)	40 (100)	
M1 ^≈^ M2	7 (25)	0	
Total	28 (100)	40 (100)	< 0.0005
**CD163+pRBP-J+ : CD163+CMAF+ cells^b^**			
M1 > M2	17 (60.7)	2 (5)	
M2 > M1	4 (14.3)	29 (72.5)	
M1 ^≈^ M2	7 (25)	9 (22.5)	
Total	28 (100)	19 (100)	< 0.0005
**CD68+pSTAT1+ : CD68+CMAF+ cells^c^**			
M1 > M2	25 (89.3)	0	
M2 > M1	1 (3.6)	40 (100)	
M1 ^≈^ M2	2 (7.1)	0	
Total	28 (100)	40 (100)	< 0.0005
**CD68+RBP-J+ : CD68+CMAF+ cells^d^**			
M1 > M2	27 (96.4)	3 (7.5)	
M2 > M1	0	25 (62.5)	
M1 ^≈^ M2	1 (3.6)	12 (30)	
Total	28 (100)	40 (100)	< 0.0005

^a^ Ratio between the numbers of CD163+pSTAT1+ macrophages (M1) and CD163+CMAF+ macrophages (M2); b) Ratio between the numbers of CD163+RBP-J+ macrophages (M1) and CD163+CMAF+ macrophages (M2); c) Ratio between the numbers of CD68+pSTAT1+ macrophages (M1) and CD68+CMAF+ macrophages (M2); d) Ratio between the numbers of CD68+RBP-J+ macrophages (M1) and CD68+CMAF+ macrophages (M2).

**Figure 3:**
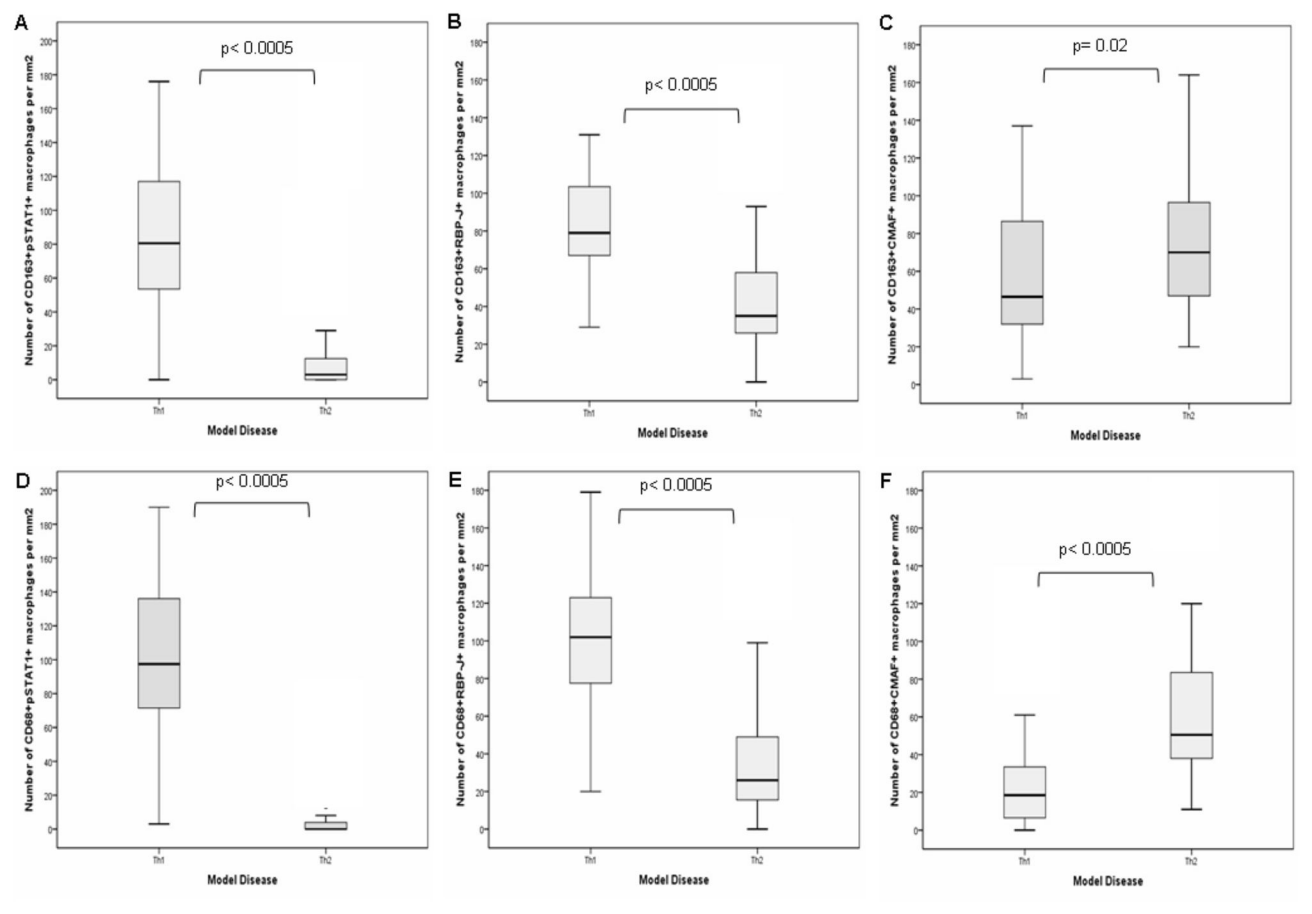
Box-plot graphs showing the numerical distribution of M1 (CD163+pSTAT1+ cells [A], CD163+RBP-J+ cells [B], CD68+pSTAT1+ cells [D] and CD68+RBP-J+ cells [E]) and M2 macrophages (CD163+CMAF+ cells [C] and CD68+CMAF+ cells [F]) according to Th model diaseases. The P-values are from Mann-Whitney tests.

To evaluate if an antibody specific for RBP-J could be used in combination with CD68 or CD163 to identify M1 polarization, we used CD68/RBP-J or CD163/RBP-J double-staining. Higher numbers of CD68+RBP-J+ macrophages were observed in the group of Th1 diseases (41 to 179; median 108 cells/mm^2^) than in the Th2 group (5 to 143; median 38 cells/mm^2^) (P< 0.0005). Similarly, higher numbers of CD163+RBP-J+ cells were present in the Th1 group (29 to 261; median 79 cells/mm^2^), while lower numbers were observed in Th2 group (3 to 111; median 42 cells/mm^2^) (P< 0.0005) (Table 1 and Figure 3). 

96.4% (27/28) of Th1 model disease cases exhibited higher numbers of CD68+RBP-J+ macrophages than of CD68+CMAF+ cells, while 62.5% (25/40) of Th2 model disease cases displayed more CD68+CMAF+ macrophages than CD68+RBP-J+ cells (M2>M1) (P< 0.0005) (Table 2). In only 1 Th1 case, similar numbers of CD68+RBP-J+ and CD68+CMAF+ macrophages (M1 ^≈^ M2) were seen. In the Th2 disease group, 12 cases (30%) showed an M1 ^≈^ M2 pattern (3 foreign body granulomas; 4 wound healing, 2 allergic nasal polyps, and 3 oxyuriasis) and 3 cases (7.5%; all foreign body granulomas) showed an M1>M2 phenotype (Supporting Information, Table S2 and Figure S1).

60.7% (17/28) of Th1 disease cases presented a predominance of CD163+RBP-J+ macrophages over CD163+CMAF+ cells (M1>M2), while 72.5% (29/40) of Th2 model disease cases displayed more CD163+CMAF+ cells than CD163+RBP-J+ cells (M2>M1) (P< 0.0005) (Table 2). 16 cases with similar numbers of CD163+RBP-J+ and CD163+CMAF+ macrophages (M1 ^≈^ M2) were observed, including 7 Th1 group disease cases (4 infectious mononucleosis, 3 Crohn’s disease) and 9 Th2 group cases (1 oxyuriasis, 2 foreign body granulomas, 3 wound healing, 3 allergic nasal polyps) (Supporting Information, Table S2).

Supporting the conclusion that RBP-J is a marker of M1 polarization in macrophages, we observed a direct correlation between CD68+RBP-J+ and CD68+pSTAT1+ macrophages (P< 0.0005), while there was no direct correlation between CD68+RBP-J+ and CD68+CMAF+ macrophages. Similarly, a direct correlation between the number of CD163+RBP-J+ and CD163+pSTAT1+ macrophages was seen (P< 0.0005), while no direct correlation was observed between CD163+RBP-J+ and CD163+CMAF+ macrophages. 

**Figure 4:**
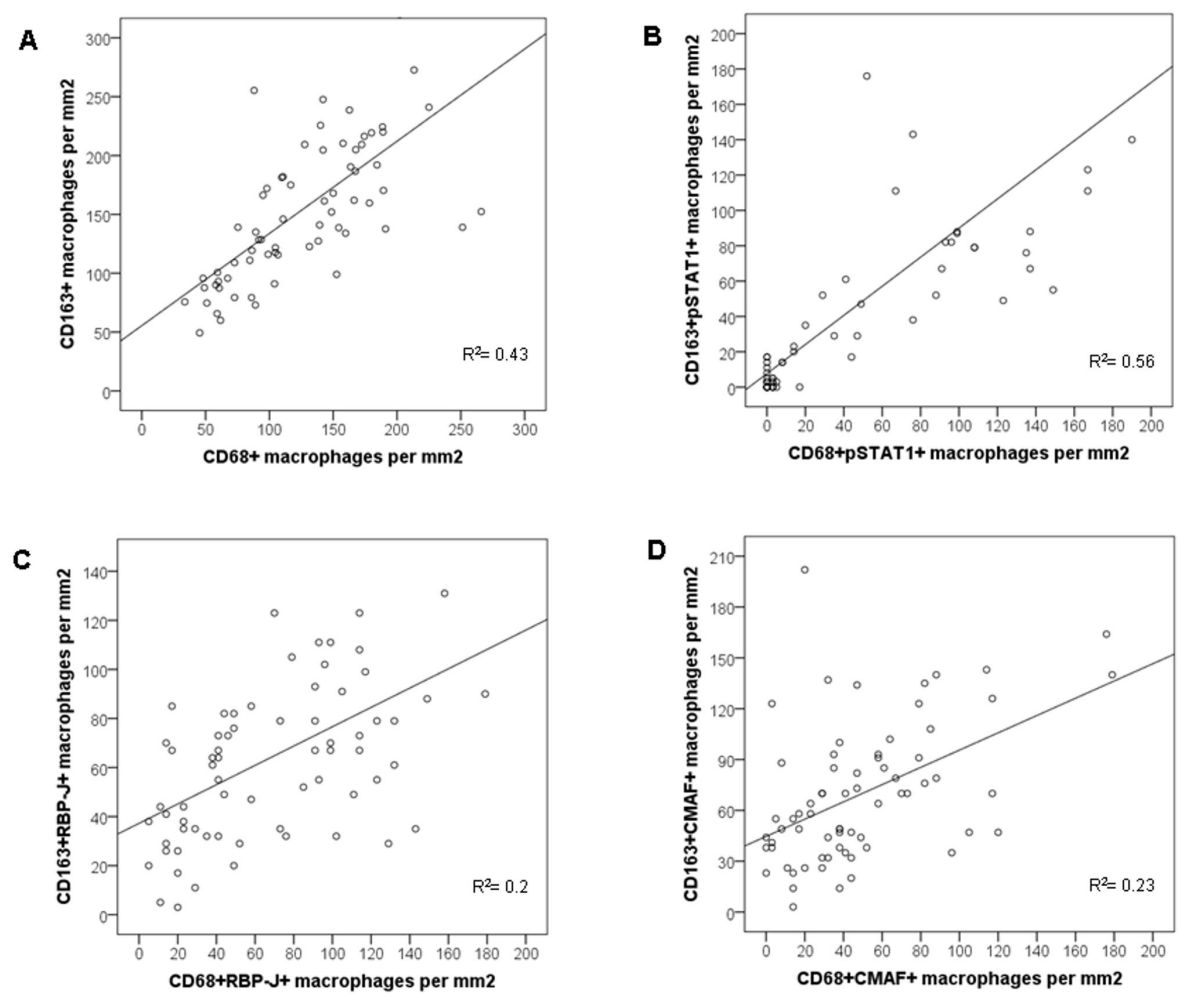
Scatter plots showing the correlation between the numbers of CD163+ and CD68+ macrophages (A), CD163+pSTAT1+ and CD68+pSTAT1+ macrophages (B), CD163+RBP-J+ and CD68+RBP-J macrophages (C), and CD163+CMAF+ and CD68+CMAF+ macrophages (D). R^2^ is the constant correlation.

When only the expression of generic macrophage markers was considered, we noticed that overall the numbers of CD163+ macrophages (from 49 to 369; median 140 cells/mm^2^) were higher than the numbers of CD68+ macrophages (from 34 to 266; median 122 cells/mm^2^) (R^2^= 0.43) (Table 1 and Figure 4).

A similar relationship was also observed when CD68 or CD163 were evaluated in the context of transcription factor expression (Figure 3). The numbers of CD163+pSTAT1+ macrophages (0 to 272, median 15 cells/mm^2^) were higher than the numbers of CD68+pSTAT1+ macrophages (0 to 190, median 8 cells/mm^2^) (R^2^= 0.56) (Figure 4 and Supporting Information, Table S2). In the same way, the numbers of CD163+CMAF+ macrophages (3 to 202, median 61 cells/mm^2^) were higher than the numbers of CD68+CMAF+ macrophages (0 to 179, median 38 cells/mm^2^) (R^2^= 0.23) (Figure 4 and Supporting Information, Table S2 and Figure S2).

Considering RBP-J, the numbers of CD163+RBP-J+ macrophages (3 to 261, median 64 cells/mm^2^) were higher than the numbers of CD68+RBP-J+ macrophages (5 to 179, median 58 cells/mm^2^) (R^2^= 0.2) (Figure 4 and Supporting Information, Table S2 and Figure S2). 

## Discussion

Polarised macrophages are characterised by the differential expression of molecules such as iNOS, metalloproteinases and arginase [1–4].. However, these antigens are not suitable as single markers for the identification of M1 or M2 macrophages as they are also expressed by other cells [1]. We, therefore, reasoned that for the characterisation of macrophage polarisation in situ, a double labelling approach would be required allowing the identification of M1- or M2-specific antigens in the context of CD68- or CD163-positive cells. Since markers such as iNOS, metalloproteinases and arginase are localised in the cytoplasm, the combined detection with CD68 or CD163 may lead to mixed colour products which may be difficult to evaluate. Because macrophage polarisation is driven by defined transcription factors, we have hypothesised that double-labelling immunhistochemistry for the combined detection of generic macrophage markers and these transcription factors may aid in the characterisation of macrophage polarisation *in situ*. There are well-established relationships between Th1 immune response and M1 polarization, as well as between Th2 response and M2 polarization [4,16,33,34]. To test our hypothesis, we have therefore analysed conditions associated with Th1- (infectious mononucleosis, Crohn’s disease) [23,24] or Th2-predominant (oxyuriasis, allergic nasal polyps, wound healing, foreign body granulomas) [2,16,25–28] immune responses. 

The preliminary data classification strategy, using hierarchical cluster analysis, highlighted patterns of association that were subsequently confirmed in the statistical analysis. As expected, the Th1 model diseases exhibited high numbers of CD68+pSTAT1+ and CD163+pSTAT1+ macrophages, while in the Th2 model diseases high numbers of CD68+CMAF+ and CDCD163+CMAF+ macrophages were observed. Similarly, a polarization in favour of putative M1 macrophages (M1 > M2) was displayed in Th1 model diseases, while a putative M2 polarization (M2 > M1) was noticed in Th2 model diseases. In view of these results and considering the known functions of pSTAT1 in regulation of the expression of interferon-stimulated genes [4,17] and of CMAF in IL10 gene expression in macrophages [18], we conclude that pSTAT1+ macrophages represent M1 macrophages, while CMAF+ macrophages represent M2 macrophages. These features support the use of CD68/pSTAT1 or CD163/pSTAT1 double-staining to identify M1 macrophages and CD68/CMAF or CD163/CMAF double-staining to identify M2 macrophages.

In addition, we evaluated the utility of RBP-J for identifying macrophage polarization in humans. As Notch signalling regulates M1 polarization and as this effect is mediated by RBP-J [21,22,35], it is plausible to assume that macrophages expressing RBP-J represent M1 macrophages. In this study, highest numbers of CD68+RBP-J+ and CD163+RBP-J+ macrophages were observed in Th1 diseases while lowest numbers were noted in the Th2 diseases. Furthermore, the Th1 model diseases exhibited polarization in favour of CD68+RBP-J+ and CD163+RBP-J+ macrophages, when compared with CD68+CMAF+ and CD163+CMAF+ macrophages, respectively. Th2 model diseases, in contrast, exhibited a higher prevalence of CD68+CMAF+ and CD163+CMAF+ macrophages when compared with CD68+RBP-J+ and CD163+RBP-J+ macrophages, respectively. Therefore, our results combined with the established biological role of RBP-J allow the conclusion that CD68+RBP-J+ and CD163+RBP-J+ cells indeed represent M1 macrophages.

Based on *in vitro* studies, it has been suggested that CD163 may be an M2-specific marker [6–8], and several studies addressing the prognostic significance of macrophages in malignancies have been conducted based on this assumption [36–39]. Our previous study of classical Hodgkin lymphoma already indicated that CD163 may not be a specific marker for M2 polarization since the numbers of CD163+ macrophages were higher in tumour microenvironment of cases with a cytotoxic/Th1 signature [15]. Here we provide further evidence that CD163 may not be M2-specific. 

Our results also disclose that CD68 (clone PG-M1) and CD163 (clone 10D6) are not equivalent in the identification of macrophages and/or macrophage polarization. Both, CD68 and CD163, are able to identify macrophages. CD163 is a specific-macrophage marker [40–42], while CD68 may also identify dendritic cell subsets [40]. In our hands, higher numbers of labelled cells where always observed using the CD163 antibody than with the CD68-specific reagent. Thus, the use of CD68 may lead to an underestimation of the true macrophage numbers. This is important particularly for cancer research, where the number of macrophages may serve as a prognostic factor [15,36–39,43]

In summary, our results show that CD68 or CD163 in combination with pSTAT1 or RBP-J can be used to identify M1 polarised macrophages, while in combination with CMAF they serve to identify M2 macrophages. Moreover, our results suggest that CD163 is not a M2-specific marker. Our results also show that in human disease conditions characterised by either Th1- or Th2-predominant immune responses differently polarised macrophages co-exist. This observation supports the notion that macrophage polarisation is a dynamic process and that the investigation of human disease processes *in situ* is important for the understanding of the role of macrophage polarisation in pathogenesis [5]. 

## Supporting Information

Table S1 **Antibodies used for immunohistochemical study.**
(DOC)

Table S2 **Balance of polarized macrophages according to diseases.**
(DOC)

Figure S1 **Box-plot graphs showing each evaluated disease according to the distribution of diseases taking into consideration the ration between CD163+CMAF+ macrophages and CD163+pSTAT1+ or CD163+RBP-J+ macrophages** (A) and CD68+CMAF+ macrophages and CD68+pSTAT1+ or CD68+RBP-J+ macrophages (B).
(TIF)

Figure S2 **Difference area charts showing the relation between CD163+pSTAT1+ and CD163+CMAF+ macrophages, taking into consideration the total number of CD163+ macrophages** (A); CD163+RBP-J+ and CD163+CMAF+ macrophages, taking into consideration the total number of CD163+ macrophages (B); CD68+pSTAT1+ and CD68+CMAF+ macrophages, taking into consideration the total number of CD68+ macrophages (C); CD68+RBP-J+ and CD68+CMAF+ macrophages, taking into consideration the total number of CD68+ macrophages (D).
(TIF)
