# Supplementary material for: Macrophage Polarisation: an Immunohistochemical Approach for Identifying M1 and M2 Macrophages
Source: PLoS One. 2013 Nov 15;8(11):e80908. doi: 10.1371/journal.pone.0080908 (PMC3829941; doi:10.1371/journal.pone.0080908)
Supplement: Table S1 — Antibodies used for immunohistochemical study. (DOC) [file pone.0080908.s001.doc]

Supporting Information (Table S1): Antibodies used for immunohistochemical study.

| **Antibody** | **Clone** | **Source** | **Dilution** | **Buffer Retrieval** |
| --- | --- | --- | --- | --- |
| pSTAT1 | Polyclonal | Santa Cruz | 1:100 | EDTA |
| CMAF | M-153 | Santa Cruz | 1:50 | EDTA |
| RBP-J | 3E2 | Dr. Kremmer | 1:8 | EDTA |
| CD68 | PG-M1 | Dako | 1:1500 | EDTA |
| CD163 | 10D6 | Novocastra | 1:3000 | EDTA |
