# Supplementary material for: Macrophage Polarisation: an Immunohistochemical Approach for Identifying M1 and M2 Macrophages
Source: PLoS One. 2013 Nov 15;8(11):e80908. doi: 10.1371/journal.pone.0080908 (PMC3829941; doi:10.1371/journal.pone.0080908)
Supplement: Table S2 — Balance of polarized macrophages according to diseases. (DOC) [file pone.0080908.s002.doc]

Supporting Information (Table S2): Balance of polarized macrophages according to diseases.

| **MACROPHAGE BALANCE** | **Infectious Mononucleosis**  **(%)** | **Crohn´s Disease**  **(%)** | **Foreign Body Granuloma**  **(%)** | **Wound Healing**  **(%)** | **Allergic Nasal Polyp**  **(%)** | **Oxyuriasis**  **(%)** |
| --- | --- | --- | --- | --- | --- | --- |
| **CD68+pSTAT1+ : CD68+CMAF+ cells^a^** |  |  |  |  |  |  |
| M1 > M2 | 15 (88.2) | 10 (91) | 0 | 0 | 0 | 0 |
| M2 > M1 | 0 | 1 (9) | 9 (100) | 10 (100) | 10 | 11 (100) |
| M1 ≈ M2 | 2 (11.8) | 0 | 0 | 0 | 0 | 0 |
| Total | 17 (100) | 11 (100) | 9 (100) | 10 (100) | 10 (100) | 11 (100) |
| **CD68+RBP-J+ : CD68+CMAF+ cells^b^** |  |  |  |  |  |  |
| M1 > M2 | 16 (94.1) | 11 (100) | 3 (33.3) | 0 | 0 | 0 |
| M2 > M1 | 0 | 0 | 3 (33.3) | 6 (60) | 8 (80) | 8 (72.7) |
| M1 ≈ M2 | 1 (5.9) | 0 | 3 (33.3) | 4 (40) | 2 (20) | 3 (27.3) |
| Total | 17 (100) | 11 (100) | 9 (100) | 10 (100) | 10 (100) | 11 (100) |
| **CD163+pSTAT1+ : CD163+CMAF+ cells^c^** |  |  |  |  |  |  |
| M1 > M2 | 12 (70.6) | 6 (54.5) | 0 | 0 | 0 | 0 |
| M2 > M1 | 2 (11.8) | 1 (9.1) | 9 (100) | 10 (100) | 10 (100) | 11 (100) |
| M1 ≈ M2 | 3 (17.6) | 4 (36.4) | 0 | 0 | 0 | 0 |
| Total | 17 (100) | 11 (100) | 9 (100) | 10 (100) | 10 (100) | 11 (100) |
| **CD163+pRBP-J+ : CD163+CMAF+ cells^d^** |  |  |  |  |  |  |
| M1 > M2 | 9 (52.9) | 8 (72.7) | 0 | 1 (10) | 0 | 1 (9) |
| M2 > M1 | 4 (23.5) | 0 | 7 (77.8) | 6 (60) | 7 (70) | 9 (82) |
| M1 ≈ M2 | 4 (23.5) | 3 (27.3) | 2 (22.2) | 3 (30) | 3 (30) | 1 (9) |
| Total | 17 (100) | 11 (100) | 9 (100) | 10 (100) | 10 (100) | 11 (100) |

a) Ratio between the numbers of CD68+pSTAT1+ macrophages (M1) and CD68+CMAF+ macrophages (M2); b) Ratio between the numbers of CD68+RBP-J+ macrophages (M1) and CD68+CMAF+ macrophages (M2). c) Ratio between the numbers of CD163+pSTAT1+ macrophages (M1) and CD163+CMAF+ macrophages (M2); d) Ratio between the numbers of CD163+RBP-J+ macrophages (M1) and CD163+CMAF+ macrophages (M2).
