# Supplementary figures and images for: Macrophage Polarisation: an Immunohistochemical Approach for Identifying M1 and M2 Macrophages
Source: PLoS One. 2013 Nov 15;8(11):e80908. doi: 10.1371/journal.pone.0080908 (PMC3829941; doi:10.1371/journal.pone.0080908)

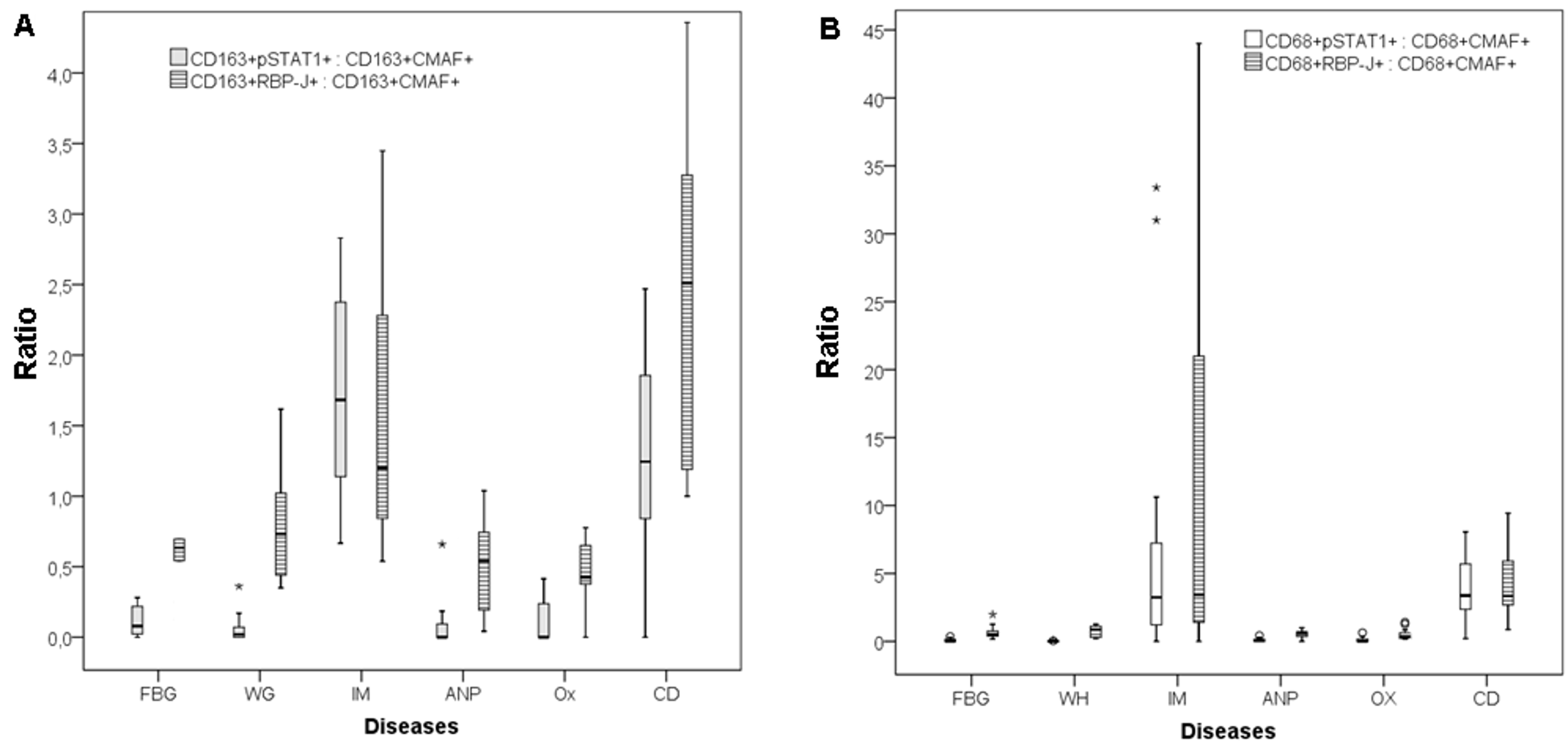

Supplement: Figure S1 — Box-plot graphs showing each evaluated disease according to the distribution of diseases taking into consideration the ration between CD163+CMAF+ macrophages and CD163+pSTAT1+ or CD163+RBP-J+ macrophages (A) and CD68+CMAF+ macrophages and CD68+pSTAT1+ or CD68+RBP-J+ macrophages (B). (TIF) [file pone.0080908.s003.tif]

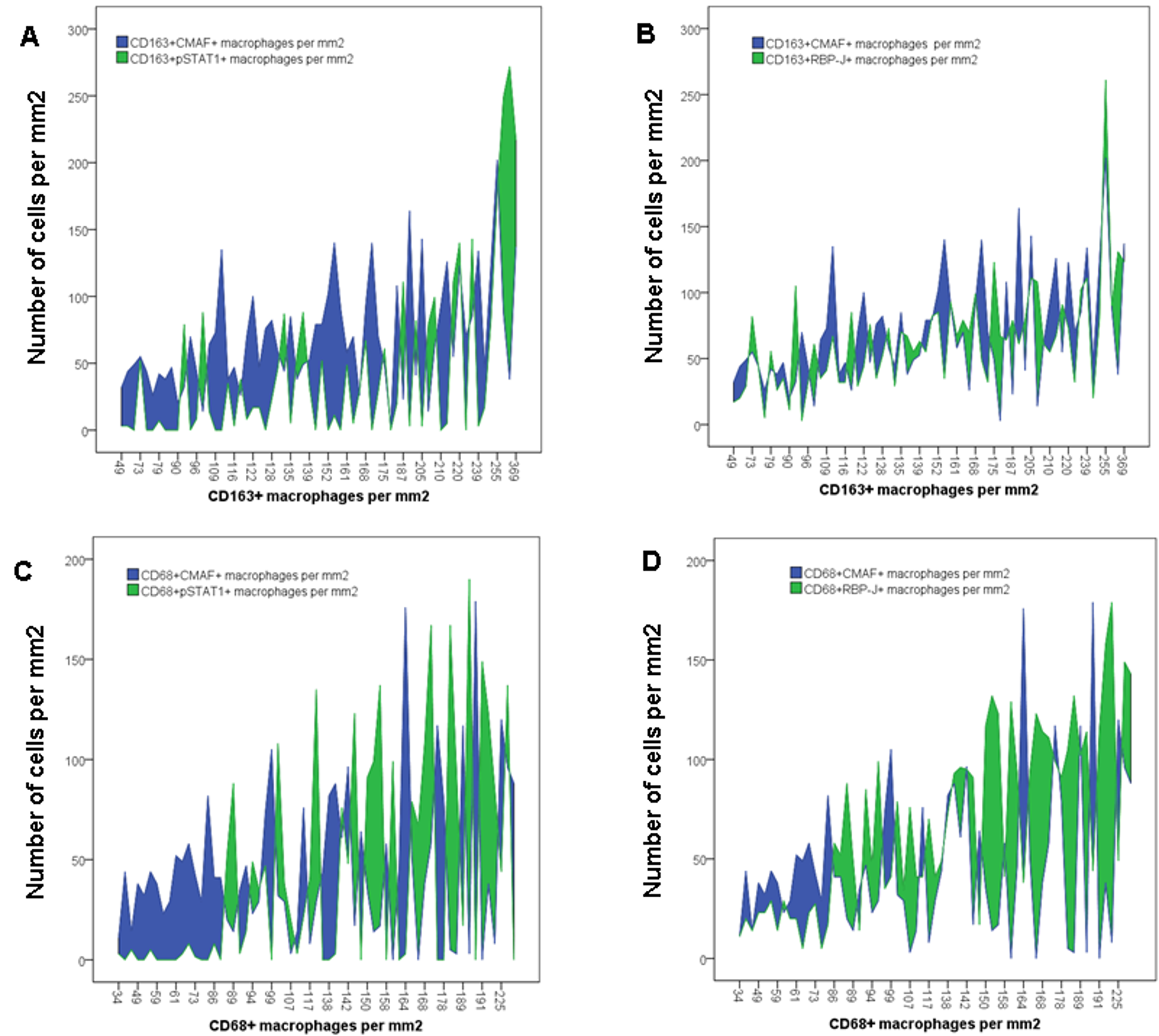

Supplement: Figure S2 — Difference area charts showing the relation between CD163+pSTAT1+ and CD163+CMAF+ macrophages, taking into consideration the total number of CD163+ macrophages (A); CD163+RBP-J+ and CD163+CMAF+ macrophages, taking into consideration the total number of CD163+ macrophages (B); CD68+pSTAT1+ and CD68+CMAF+ macrophages, taking into consideration the total number of CD68+ macrophages (C); CD68+RBP-J+ and CD68+CMAF+ macrophages, taking into consideration the total number of CD68+ macrophages (D). (TIF) [file pone.0080908.s004.tif]
